# Endophytic Fungal Consortia Enhance Basal Drought-Tolerance in *Moringa oleifera* by Upregulating the Antioxidant Enzyme *(APX)* through *Heat Shock Factors*

**DOI:** 10.3390/antiox11091669

**Published:** 2022-08-27

**Authors:** Javeria Javed, Mamoona Rauf, Muhammad Arif, Muhammad Hamayun, Humaira Gul, Aziz Ud-Din, Jalal Ud-Din, Mohammad Sohail, Muhammad Mizanur Rahman, In-Jung Lee

**Affiliations:** 1 Department of Botany, Abdul Wali Khan University Mardan, Mardan 23200, Pakistan; 2 Department of Biotechnology, Abdul Wali Khan University Mardan, Mardan 23200, Pakistan; 3 Department of Biotechnology and Genetic Engineering, Hazara University Mansehra, Mansehra 21120, Pakistan; 4 Department of Biotechnology and Genetic Engineering, Islamic University, Kushtia 7003, Bangladesh; 5 Department of Applied Biosciences, Kyungpook National University, Daegu 41566, Korea

**Keywords:** plant-microbe interaction, *Moringa oleifera*, drought stress, phytohormones, antioxidants, ascorbate peroxidase, *heat shock factors*, hydrogen peroxide

## Abstract

Global climate change has imposed harsh environmental conditions such as drought. Naturally, the most compatible fungal consortia operate synergistically to enhance plant growth and ecophysiological responses against abiotic strains. Yet, little is known about the interactions between phytohormone-producing endophytic fungal symbionts and plant growth under drought stress. The existing research was rationalized to recognize the role of newly isolated drought-resistant, antioxidant-rich endophytic fungal consortia hosting a xerophytic plant, *Carthamus oxycantha* L., inoculated to *Moringa oleifera* L. grown under drought stress of 8% PEG (polyethylene glycol-8000). Under drought stress, the combined inoculation of endophytic strain *Microdochium majus* (WA), *Meyerozyma guilliermondi* (TG), and *Aspergillus aculeatus* (TL3) exhibited a significant improvement in growth attributes such as shoot fresh weight (1.71-fold), shoot length (0.86-fold), root length (0.65-fold), dry weight (2.18-fold), total chlorophyll (0.46-fold), and carotenoids (0.87-fold) in comparison to control (8% PEG). Primary and secondary metabolites were also increased in *M. oleifera* inoculated with endophytic consortia, under drought stress, such as proteins (1.3-fold), sugars (0.58-fold), lipids (0.41-fold), phenols (0.36-fold), flavonoids (0.52-fold), proline (0.6-fold), indole acetic acid (IAA) (4.5-fold), gibberellic acid (GA) (0.7-fold), salicylic acid (SA) (0.8-fold), ascorbic acid (ASA) (1.85-fold), while abscisic acid (ABA) level was decreased (−0.61-fold) in comparison to the control (8% PEG). Under drought stress, combined inoculation (WA, TG, TL3) also promoted the antioxidant activities of enzymes such as ascorbate peroxidase (APX) (3.5-fold), catalase (CAT) activity (1.7-fold), and increased the total antioxidant capacity (TAC) (0.78-fold) with reduced reactive oxygen species (ROS) such as H_2_O_2_ production (−0.4-fold), compared to control (8% PEG), and stomatal aperture was larger (3.5-fold) with a lesser decrease (−0.02-fold) in water potential. Moreover, combined inoculation (WA, TG, TL3) up regulated the expression of *MolHSF3, MolHSF19,* and *MolAPX* genes in *M. oleifera* under drought stress, compared to the control (8% PEG), is suggestive of an important regulatory role for drought stress tolerance governed by fungal endophytes. The current research supports the exploitation of the compatible endophytic fungi for establishing the tripartite mutualistic symbiosis in *M. oleifera* to alleviate the adverse effects of drought stress through strong antioxidant activities.

## 1. Introduction

Plants are sessile, hence challenged by several environmental complications that convert further critical as abiotic stresses including low temperature, high salinity, and drought progressively restrict plant development and crop yields [1,2]. Through the evolutionary process, plants have evolved defensive mechanisms (morphological, physiological, and molecular plasticity and adaptability) against stresses [3]. Stress severity, duration, speed, and recovery efficacy all play a role in regulating plant performance during severe water scarcity periods, which rely on plant-genotype-specific traits [4]. Water deprivation stress is one of these limiting environmental constraints that has received a lot of attention since it is likely the principal restriction for agricultural yield in many dry and semi-arid places throughout the world. Plants must adapt fast in the event of water shortage; hence water deficit circumstances affect almost all biological activities at the whole plant level [5]. Hyperarid zones cover 6.6% of the earth’s land area, arid zones cover 10.6%, semiarid zones are more widespread, present on all continents, and cover 15.2%, and dry subhumid zones cover 8.7% [6].

Drought is a serious hazard and the most unexpected limitation, affecting agricultural output globally. It impacts practically all elements of the plant, from germination to maturity [7]. Drought is a severe danger to the viability of crop growing regimes across the world due to rapidly changing climatic dynamics and underwater restricting conditions, plant height, leaf size, and stem girth were all also considerably reduced [8]. Drought stress causes a drop in relative water content, a fall in leaf water potential, turgor loss, and cell enlargement reduction, followed by a decrease in photosynthetic pigments, disruption of several metabolic processes, ROS burst, and eventually plant death [9]. One of the stress-reduction mechanisms is the hyper-activation of the ROS scavenging machinery, which helps to avoid cell damage and restore redox balance. The expression and protein level of ROS scavenging genes is raised during stress in many different plant species and has been linked to baseline stress resistance. Furthermore, the activation of scavenging genes was substantially greater in stress-tolerant genotypes than in sensitive ones and boosting antioxidant enzyme activities improved plant drought stress resistance. ROS scavenging is crucial in the drought-stress response, where both the enzymatic and non-enzymatic antioxidants, such as superoxide dismutase (SOD), ascorbate peroxidase (APX), and catalase (CAT), are important in the detoxification of ROS [10,11,12,13]. 

It is unavoidable for plants to resist the drought stress caused by arid environments. Drought-tolerant plants are known as xerophytics because they have developed methods to withstand drought stress, such as: (i) Biosynthesis and accumulation of osmolytes to control turgor pressure that prevents structural membrane damage, regularize ionic balance and homeostasis, modulate water use efficiency and uptake; (ii) flexibility in overall growth and its plasticity (morpho-physiological pattern change); (iii) increased photosynthetic activity; (iv) accelerated antioxidant potential for ROS scavenging; (v) phytohormones biosynthesis; (vi) growth-promoting and stress mitigating metabolic production; and (vi) genetic adaptability to modulate the gene expression controlling stress perception and cell signaling along with the induction of gene expression for metabolite and hormonal accumulation to trigger the stress alleviation process, under drought stress. Nevertheless, these adaptations are not adequate to trigger resistance against drastically prevailing drought worldwide, and not all plant species have the complete ability to cope with the drought stress. Therefore, drought stress has been found to negatively affect the growth and development of *M. oleifera* as well [14,15]. According to researchers, *M. oleifera* is a promising plant species since it is a medicinally and nutritionally rich plant commonly known as drumstick tree, that can absorb 20 times more carbon dioxide than other flora. The moringa tree, which has been successfully rehabilitated to reduce the negative impacts of drought, may also be utilized for water purification and soil conservation in agroforestry systems in arid and dryland locations across the world [16]. Worldwide demands for food entail the cultivation of drought stress-resistant, medicinally, and nutritionally important plants such as *M. oleifera* [17]. *M. oleifera* grown under water stress of −1.5 MPa triggered by polyethylene glycol (PEG-6000) showed a positive stress resistance response but the overall growth and development were compromised compared to control [18]. Keeping in view the importance of *M. oleifera* as a global miracle tree, the current study was rationalized to enhance the drought stress resistance of *M. oleifera* with better growth performance.

Apart from morpho-physiological modifications, numerous gene expression investigations had been performed to understand the drought-responsive mechanisms in various crops but in *M. oleifera*, it has not been explored yet. Therefore, researchers have initiated to exploit different strategies that might be useful to ameliorate the drought stress tolerance of *M. oleifera*. High-quality genome sequencing appears to be a potent tool for improving the desired growth traits of plants as well as exploring the molecular bases for activation of stress tolerance-associated pathways to improve the stress resistance efficiency in plants. High-quality genome sequencing and comparative genomic analysis in *M. oleifera* have allowed researchers to discover the inducible stress-resistant genes [19]. More recently, another report on *M. oleifera* transcriptome analysis, along with qRT-PCR has enabled the identification of stress-responsive transcription factors [20]. More recently, whole-genome sequencing (WGS) of *M. oleifera* has been performed that aided the identification of several ion channel transporters, chaperons, penta-tricopeptide proteins, and various transcription factors (*HSFs*, *WRKY*, and *CCCH* zinc finger) important for the survival of plants under stress. qRT-PCR also confirmed that *MolHSF-8* upregulated in the *M. oleifera* roots the first tissue that senses drought stress to respond faster, signifying a critical regulatory role during drought stress [21]. *HSFs* participate to control the response to several other stresses as well, including cold, heat, and salinity [22]. Another strategy adopted by scientists to induce adverse drought resistance in *M. oleifera* is the application of exogenous priming mediators such as trehalose [14], and melatonin [15]. However, the usage and production of these compounds are laborious, costly, and in most cases harmful to the environment. Additionally, the effectiveness of synthetic fertilizers, chemical-based priming mediators, as well as growth regulators vary with environmental stress and plant type. Another well-known technique for mitigating the damaging effects of abiotic stressors on crops is to use extreme-habitat-adapted symbiotic microbes. Drought and other extreme environmental circumstances have raised the possibility of discovering fungal endophytes capable of establishing drought stress tolerance in non-host plants. Scientists, therefore, have adapted this alternative promising approach of utilizing a low-cost, eco-friendly, and non-toxic biological source such as endophytic fungi, for inducing drought-stress tolerance along with efficient growth promotion through modulation in phytohormonal production and moisture-holding capacity of plants.

Fungal microbes residing inside the plants have been shown little or no harm or disease signs in their hosts. Endophytes are found in almost every vascular plant studied so far, and they are thought to protrude from the plant parts of phyllospheric and rhizospheric regions and intrude the host plant through wounded areas or stomatal openings. Much research has been conducted in the recent years to investigate the endophytic communities associated with various plant species. Endophytic microbes use a variety of ways to enhance plant development and guard against pests and diseases. Endophytes produce and release secondary metabolites/biochemicals that inhibit or minimize the harmful effects of plant diseases, such as volatile substances that inhibit pathogen development. Other endophytes defend their host plants by triggering plant defense mechanisms, which can be accomplished by either systemic acquired resistance (SAR) or induced systemic resistance (ISR) [23]. Moreover, endophytic microorganisms that promote plant development are also known for their role as drought-tolerance enhancers, which show a variety of strategies for dealing with the effects of drought on plants and soil through modulating the physiological, biochemical, and molecular changes in the plant. These microbes influence plants through the following mechanisms: (1) Alteration in phytohormonal activity; (2) generation of aminocyclopropane-1-carboxylate deaminase (ACCd) to minimize ethylene levels in roots; (3) deposition of osmolytes that impart drought resistance in plants; (4) exopolysaccharide (EPS) manufacturing; (5) microbial volatile organic compounds (mVOCs) generation; (6) antioxidant defense [24].

The current study, therefore, was focused to explore the effect of individual endophytic strains *Microdochium majus* (WA), *Meyerozyma guilliermondi* (TG), *Aspergillus aculeatus* (TL3), and a combination of these strains as a consortium (WA+TG+TL3) in *M. oleifera* under PEG-8000 induced drought stress. The outcome of recent research permitted us to unravel the detailed biochemical, physiological, metabolic, antioxidant, and phytohormonal rebalancing for the enhancement of drought stress tolerance *M. oleifera.*

## 2. Materials and Methods

### 2.1. Endophytic Fungal Isolation, Characterization, and Identification

#### 2.1.1. Isolation and Purification of Endophytic Fungi

**Figure 1:**
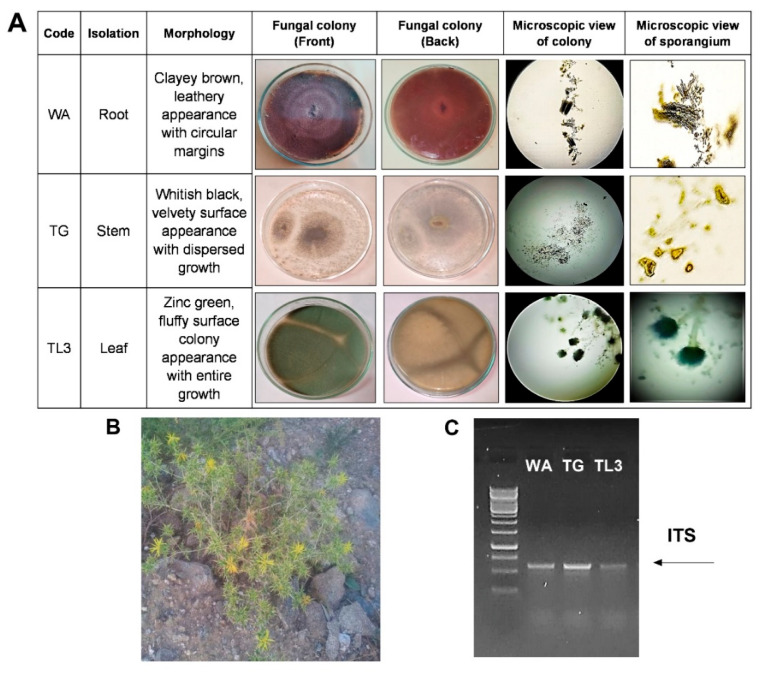
(**A**) Morphological characterization of selected fungal strains WA, TG, and TL3, (**B**) Xerophytic plant (*Carthymus oxycantha* L.) selected for isolation, and (**C**) genotyping of selected fungal strains by ITS region amplification.

For the isolation of endophytic fungi, *Carthamus oxyacantha* L. plant was selected (Figure 1). Plant samples (root, leaves, and stem) were properly collected from the dry region of Shenki Jalala area, Takht Bhai located at (34°20’8 N 71°54’10 E) an altitude of 339 m (1115 feet), Khyber Pakhtunkhwa Pakistan. Plant samples were surface sterilized (70% ethanol for 2 min, 1% perchloric acid for 30 s). To eliminate perchloric acid and ethanol residues, final washing was done with double-distilled water after sterilization. Similarly, sterilized plant parts were sliced into small pieces and 12 parts per plate were deposited on Hagem minimum media plates. The fungal colonies that had developed from plant parts were carefully picked and cultivated on potato dextrose agar (PDA) media plates for incubation (25 °C). After 7 days of incubation, various colonies of 15 endophytic fungal strains with different morphological features were taken under consideration. Each strain was carefully sub-cultured on PDA plates to get their respective pure culture. Among all, eight (8) fungal strains were properly isolated and purified on the PDA medium and then stored in the refrigerator for further usage.

#### 2.1.2. Fungal Strains Microscopy

For fungal strain microscopy, a small piece was removed, and a water drop was placed on the microscope slide and shielded with a coverslip. Gentle pressure was subjected to the coverslip to help to flatten the specimen. Mycelium was visualized at the magnification, i.e., 40× and 100× under the light microscope (Binocular NSL-CX23 Olympus, Tokyo, Japan). Lactophenol cotton blue reagent was used as a staining and mounting medium for examining the fungal colonies using the method as described [25].

#### 2.1.3. Strain Identification

The chosen endophytic fungal strain was recognized molecularly using universal primers ITS-1 and ITS-4 to amplify their ITS region of 18S rDNA. About 30 μL reaction mix was prepared for PCR using template genomic DNA (20 ng), and the cloned PCR fragments (Figure 1) were sequenced by BGI Co., Ltd. (Shenzhen, China) using the ITS-1 and ITS-4 genes, as described before [26]. By combining the reverse and forward reads with a coding codon aligner (version 7.2.1, Codon code corporations), the resulting sequences were subjected to a BLAST query in the NCBI (http://www.ncbi.nlm.nih.gov/BLAST, accessed on 20 October 2021) database to find the closest homolog sequence matches. On MEGA 7 (version 7.0.18), the closest matches were further evaluated to create individual phylogenetic trees for strain identification. Sequences were submitted to NCBI GenBank under accession numbers. ON202840, ON202837, and ON387621.

#### 2.1.4. Screening of Fungal Endophytes in PEG (Polyethylene Glycol) Containing Czapek Medium

All purified strains were selected for the preliminary screening upon drought stress induced by various concentrations of PEG-8000 (0, 2, 4, 6, and 8%), as osmoticum to mimic the osmotic pressure of fungal cells in czapek medium (50 mL). The flasks were kept in the shaking incubator at 120 rpm for seven days at 30 °C and the growth rate was checked. Similarly, after seven days, the fresh biomass/flask of each 50 mL culture (0 and 2–8% PEG) was weighed with the help of electrical balance. Among all endophytic fungi, the three strains labeled as, WA, TG, and TL3 were selected as drought stress-resistant under 8% PEG (drought stress). Drought stress-tolerant mycelia of fungi were filtered from media. Endophytic fungi produce several metabolites to cooperate with the host plant species for growth enhancement. In this context, plant hormone (IAA, GA, ABA, and SA), primary metabolites (proteins, sugars, lipids), secondary metabolites (phenolics, flavonoids, and proline), and antioxidants (enzymatic and non-enzymatic) were assessed in fungal culture. The final concentration of spore suspension (∼5 × 10^7^ spores/mL) was maintained for plant inoculum preparation. Biomass and supernatant were separated via sterilized Whatman filter paper. The pellet was reproduced by centrifuging the culture (Sartorius Modle: 2–16 PK), which was then utilized to quantify the metabolites listed. Supernatants were then frozen and kept in a −80 °C refrigerator.

#### 2.1.5. Analysis of the Endophytic Fungal Culture Filtrate

The endophytic fungal culture filtrate (7 days old culture) was retrieved by centrifugation to determine the metabolites. Flavonoids and phenols were estimated in culture filtrate by the procedure as described [27]. In brief, 0.8 mL of Folin–Ciocalteu reagent (Sigma Aldrich, Burlington, MA, USA) and 2 mL of 7.5% Na_2_CO_3_ (sodium carbonate (Sigma Aldrich, Burlington, MA, USA) were mixed with 0.2 mL of culture supernatant. The sample was diluted in dH_2_O to 7 volumes and incubated in the dark for 2 h. To create a standard curve, different concentrations of catechol were utilized. At 650 nm, the absorption was measured against a blank. Catechol concentrations (1–10 mg) were utilized to create a standard graph (CellMark AB, Göteborg, Sweden). Absorbances were recorded by using a spectrophotometer (UV/VIZ spectrophotometer; PerkinElmer Inc. United States). Primary and secondary metabolites were quantified as i.e., total soluble sugar content [28], total proteins [29], total lipids [30], proline content [31]. The estimation of indole-3-acetic acid (IAA) in the fungal filtrate was carried out using the Salkowski reagent [32]. Absorbance was measured at 540 nm. Various known concentrations (10–100 µg) of IAA (Sigma Aldrich, Burlington, MA, USA) were utilized for the calibration curve; gibberellic acid, abscisic acid [33], salicylic acid [34], and ascorbic acid [35]. Oxidative enzymes, i.e; catalase [36], total antioxidants [37], peroxidase activity [38], hydrogen peroxide [39] were also quantified in the culture filtrate.

### 2.2. Application of Endophytic Fungi on M. oleifera

#### 2.2.1. In Vitro Drought Stress Tolerance Bioassay and Field Experiment of *M. oleifera*

*M. oleifera* L. seeds were collected from National Agricultural Research Centre (*NARC*), Islamabad (33°41′35″ N 73°03′50″ E), *Pakistan.* Seed surface sterilization was done for 1 min in ethanol (70%), followed by sodium hypochlorite (20%) for 30 min. The stratified seeds were placed in the Petri plates containing wet sterilized filter papers. The seeds were imbibed and kept for 6 days at 4 °C. For checking the stress induction level of PEG-8000, different concentrations of PEG (i.e., 0%, 2%, 4%, 6%, and 8%) were applied to *M. oleifera.* To evaluate the response of *M. oleifera* toward PEG-8000 induced osmotic/drought stress under a natural environment, the seedlings of uniform germination were shifted to autoclaved soil pots (1 seed/pot) pre-mixed with WA, TG, and TL3 fungal biomass (3 g/pot). Whereas control pots lacked active culture biomass and culture filtrate. About 14 days after germination, the spore suspension of endophytic fungi was inoculated at 1 mL/seedling of each pot. Spore suspension density was adjusted to ∼5 × 10^7^ spores/mL. About 21 days after germination, 3 mL of PEG-8000 (8% osmoticum solution) was applied on alternative days, for 15 days, to the base of uniformly grown *M. oleifera* seedlings for drought stress induction. The 21-d-old seedlings were exposed to drought stress (8% PEG-8000, 3 mL/pot, 6 days) and were imperiled to a recovery period (9 days). The arrows indicate the time points of specific analysis. The *M. oleifera* plant bioassay was performed in a completely randomized design (CRD). The experimental set-up comprised of three biological replicates and each replicate comprised 15 pots (6 cm × 8 cm in diameter and height) with 1 plantlet/pot (total = 1 × 15 × 3 = 45 replicates/treatment). Pots were having a basal outlet (for leaching purposes) and 400 g of sterilized sand/soil (8/2, *v/v*) for each treatment. The plants were cultivated under field conditions in the natural environment (March 2021–May 2021, with temperatures ranging from 20 ± 2.4 °C to 37 ± 3.2 °C), in the Botanical Garden, Abdul Wali Khan University Mardan, (34°11’54” N, 72°2’45” E). Various treatments used in the current study have been listed in Supplementary Table S1.

#### 2.2.2. Analysis of Growth Attribute and Stomatal Aperture

Various growth attributes of *M. oleifera* including seed fresh weight (g), cotyledon length (cm), root and shoot length (cm), and fresh and dry weight (g) were taken into consideration for the assessment of growth response. Analysis of leaf water potential was performed as mentioned earlier [40] using the following formula

Ψ_s_ = −miRT

m = molality (moles/1000 g)

i = ionization constant (1.0 for sucrose)

R = gas constant (0.00831-L MPa mol^−1^ K^−1^)

T = Temperature in degrees K (C + 273 = K)

For stomatal anatomy, the peels were obtained from the abaxial surface of leaves and allowed to float on distilled water for 2 h under continuous illumination. The peels were then immersed in distilled water with a pH of 5.5, and the stomata were observed using a light microscope at a magnification of 100× (Binocular NSL-CX23 Olympus, Tokyo, Japan). Stomatal pictures were taken using a digital camera (Canon, Tokyo, Japan). ImageJ (http://fiji.sc/, accessed on 11 January 2022) was used to calculate the stomatal aperture, which was calibrated with a µm ruler.

#### 2.2.3. 3,3-Diaminobenzidine (DAB) Activity

By using the 3,3-diaminobenidine (DAB) assay H_2_O_2_ production and accumulation was detected through the method as described [41]. Plants parts of 1 cm in length of the 3rd leaf from the 35 days old seedlings were in-filtered in a vacuum with DAB staining solution. The incubation of samples was done in 90% ethanol at 70 °C for 10 min, to remove chlorophyll contents. To evade auto-oxidation, the working solution of DAB was freshly prepared [42]. Similarly, owing to DAB polymerization, H_2_O_2_ was viewed as a brown color, and the leaf segment photos were subsequently taken.

#### 2.2.4. Biochemical Analysis

Total chlorophyll contents were determined as described [43], in the seedlings of *M. oleifera.* Leaf samples were ground in 3 mL of 80% acetone. The total soluble sugar contents in the seedlings of *M. oleifera* were determined as described [28] by using 0.5 g of fresh plant tissue. Following the method described as [29], the protein content was estimated by using 1 g of leaves samples of *M. oleifera* by following the protocol from [30], and total lipids were determined in 1 g of fresh leaf tissue. Following the procedure as described [31], proline content in *M. oleifera* was determined by using 2 g of fresh leaf tissue. Procedure as described [44] was used for the determination of flavonoid contents and total phenolics in the *M. oleifera* by using 1 g of fresh leaf tissue.

#### 2.2.5. Determination of Phytohormones in *M. oleifera*

Following the procedure as described [9], the indole-3-acetic acid (IAA) content was determined by taking 0.5 g of crushed plant material in 5 mL distilled water, centrifuged at 10,000 rpm for 15 min, and the supernatant (1 mL) was mixed with 2 mL of Salkowski reagent (1 mL of solution A: 0.5 M ferric chloride and 50 mL solution B: 35% perchloric acid). The reaction was incubated for 30 min in the dark at room temperature. Salkowski reagent (4 mL) was used as a control. Optical density was taken at 540 nm using UV/VIZ spectrophotometer (PerkinElmer Inc., Waltham, MA, USA). For the determination of gibberellic acid (GA) and abscisic acid (ABA), the procedure of [33] was followed briefly describing the mixing of 0.5 g of crushed plant material in 60 mL of methanol: chloroform: 2N ammonium hydroxide (12:5:3 *v/v/v*), PH was adjusted to 2.5, followed by extraction procedure (thrice) by using ethyl acetate (15 mL), followed by the addition of dH_2_O (25 mL) to each sample. Two phases were formed, chloroform phase was discarded, water phase was collected, PH was adjusted to 2.5, followed by extraction procedure (thrice) by using ethyl acetate (15 mL), incubation was done for 1 h at 70 °C to isolate free form of ABA and GA. Finally, the evaporation was performed at 45 °C and methanol (2 mL) was added for elution. Optical density was taken at 254 nm for GA and 263 nm for ABA through spectrophotometer. The SA content was determined using the method of [34], which involved adding a 1:1 volume of 0.1 percent aqueous solution of iron trichloride (FeCl_3_) into an aqueous suspension of powdered 0.5 g plant tissue samples, followed by centrifugation at 4000 rpm for 10 min. The supernatant was used to compare the SA content to a known amount of pure SA standard solutions (100–1000 g/mL) e made using >99% pure SA (Sigma-Aldrich) (St. Louis, MO, USA), and the absorbance was determined spectrophotometrically at 540 nm (Thermo Spectronic, Waltham, MA, USA).

#### 2.2.6. Quantification of Catalase Activity

The procedure as described [36], was followed to quantify catalase (CAT activity) by taking 0.2 g of fresh leaves samples ground in 2 mL phosphate buffer. The supernatant was separated after centrifugation (at 10,000 rpm for 5 min) and used for catalase determination. Absorbance was measured at 240 nm after 30 s intervals (extinction-coefficient = 0.036 mM/cm). In the procedure as described [35], the ascorbate peroxidase (APX) activity was determined in fresh plant material (0.2 g) by mixing 0.1 mL enzyme extract and substrate solution (0.2 mM ascorbic acid, 50 mM potassium phosphate buffer, 0.2 mM EDTA, 20 mM H_2_O_2_). Absorbance was measured at 290 nm and the reduction in optical density was monitored every 30 s for up to 7 min.

#### 2.2.7. Multivariate Analysis

The principal components analysis (PCA) gathered all endophytic fungi, plant, and drought variables. The principal component analysis (PCA) was accomplished by using OriginPro8 software. To analyze the multivariate effect of drought on endophytic fungal traits (primary, secondary metabolites, enzymatic and non-enzymatic antioxidants, biomass production) and the effects of drought + endophytic fungal variables (WA, TG, and TL3) on plant traits (primary, secondary metabolites, enzymatic and non-enzymatic antioxidants, fresh/dry weight, stomatal aperture, water potential), the PCA was performed, separately.

#### 2.2.8. Stem Anatomical Evaluation

A free-hand sectioning technique was used to prepare permanent slides of stem transverse sections cut with the razor blade, and some fine sections were carefully picked up on wash glass for staining. For staining, the lignified tissues (xylem vessels and sclerenchyma) were transferred to safranin (1.0 g dissolved in 100 mL, 70% alcohol) for 20 min, dehydrated in 90% alcohol for 5 min. The sections were mounted in Canada balsam by putting a drop of resin on a slide and placing the sections on the slides and visualized at the magnification of 40x under the light microscope (Binocular NSL-CX23 Olympus, Tokyo, Japan).

### 2.3. RT-qPCR Analysis for Gene Expression

RT-qPCR analysis was performed as described earlier [45]. Total RNA was retrieved from 35-d-old plants (leaf and root tissue) using the GeneJET Plant RNA Purification Kit (Thermo Scientific™, Waltham, MA, USA). About 2 μg of total DNAase-treated RNA was taken for reverse transcription reaction using the RevertAid First Strand cDNA Synthesis Kit (Invitrogen, Karlsruhe, Germany). Expression analysis of selected *HSFs* and *APX* genes was performed as described [26]. The expression was normalized with the housekeeping gene *actin* as mentioned earlier [21], for the stable expression under drought stress in *M. oleifera*. Primer sequences have been given in Supplementary Table S2.

### 2.4. Statistical Analysis

The experiment was done in a completely randomized design (CRD). GraphPad Prism 9.0.0 (121) software was used to analyze the data statistically that denote the means and standard errors of three independent replicates for each treatment, using RM two-way analysis of variance (ANOVA). The statistical data were further verified by using the statistical software package SPSS V. 21.0 (SPSS, Chicago, IL, USA). Similarly, mean separation was carried out by applying Duncan’s multiple range test (DMRT). Significant differences were represented through various statistical bars marked with significant letters at *p* ≤ 0.05. Fold changes were computed as the ratio of the changes between the mean value of treated samples (mT) and the mean value of respective control samples (mC) over the mean value of respective control samples (mC), as indicated in the formula given; (mT − mC)/mC).

## 3. Results

### 3.1. Endophytic Fungal Isolation

A total of fifteen strains of endophytic fungi were isolated from the various parts of the xerophytic plant *Carthamus oxyacantha* L. (Figure 1; Supplementary Table S3).

### 3.2. Assessment of Isolated Strains for Drought Stress Tolerance

**Figure 2:**
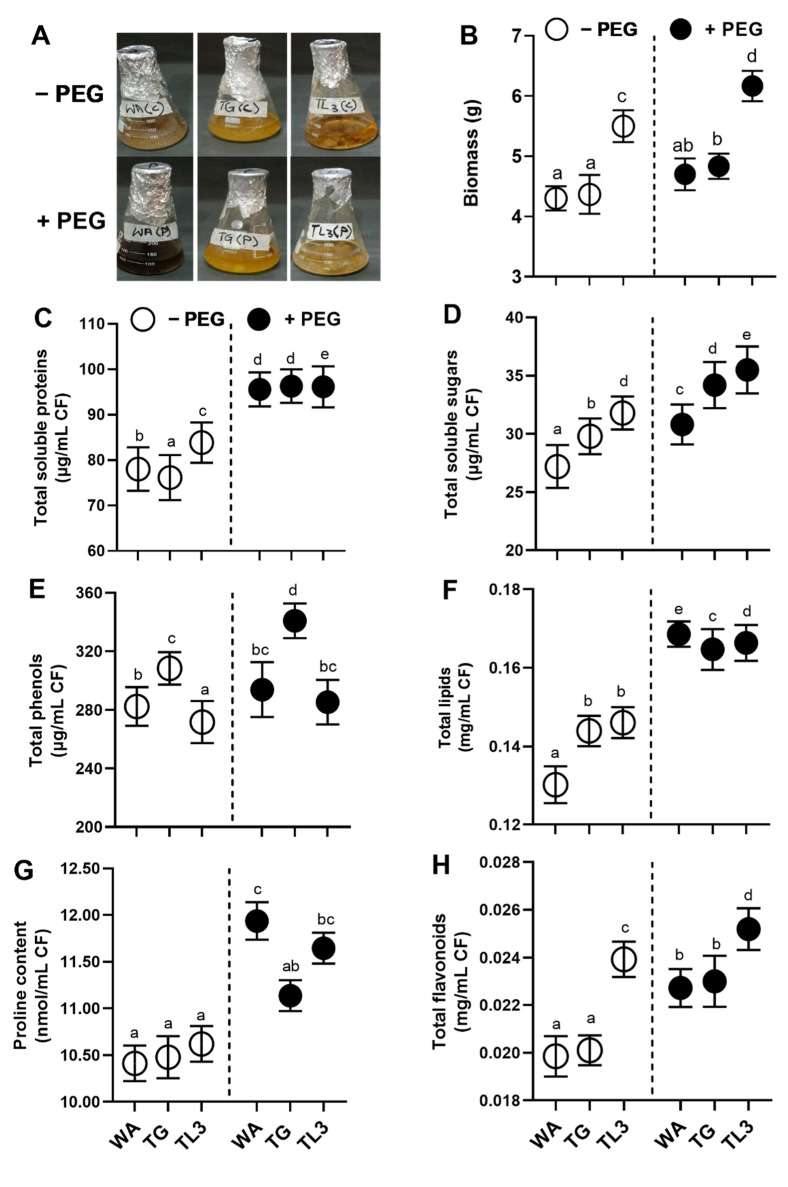
Characterization of fungal isolates. (**A**) Screening of fungal strains with 8% PEG in czapek media compared to control (0% PEG). (**B**) Biomass, (**C**) total soluble proteins, (**D**) total soluble sugars, (**E**) total lipids, (**F**) total phenols, (**G**) total flavonoids, and (**H**) proline content measured in CF of fungal isolates. Quantitative data represent means ± SE of three independent biological replicates presented with various letters showing differences at a significance level of *p ≤* 0.05 using Duncan’s multiple range test (DMRT). CF; culture filtrate.

The resistant strains against PEG-8000 (8%) were evaluated by growing on czapek medium supplemented with PEG and were allowed to grow at shaker (120 rpm, 30 °C, 7 days) and the growth of isolates was evaluated in terms of fresh weight by harvesting the fungal biomass from culture through filtration. The resistant strains were then checked for various growth parameters and applied to *M. oleifera* under PEG-mediated drought stress. Among all the isolated strains, the WA, TG, and TL3 proved tolerant by producing the highest biomass in control (0% PEG) as well as the stressed condition (8% PEG) and were selected for further experiments based on resistance response to the PEG-induced drought stress. Based on visual features, preliminary identification of chosen strains (WA, TG, and TL3) of fungal endophytes was performed. Based on the texture and shape of the colony, growth pattern, hyphae color, and spores, the fungal isolates were recognized to at least the genus level (Figure 2).

### 3.3. Molecular Identification Based on ITS Sequences and Phylogenetic Analysis

The 18 S rDNA gene amplification (Figure 1) and sequencing were performed for WA, TG, and TL3 isolates. Following sequencing, the identified sequence was matched to the data in the GenBank sequence database to determine the genus or species of WA, TG, and TL3 isolates. Phylogenetic relationships have been shown in Supplementary Figure S1. The sequences shown in this study can be retrieved from the online repository. The ITS sequence of WA was classified to the species level after a homology search on GenBank that revealed 100% similarity to *Microdochium majus.* Hence the strain was identified and named *Microdochium majus.* The sequence was submitted to NCBI GenBank under accession No. ON202840. Similarity ITS sequence of TG showed 100% similarity with *Meyerozyma guilliermondi*, so the TG strain was identified and named *Meyerozyma guilliermondi.* The sequence was submitted to NCBI GenBank under accession No. ON202837. While the ITS region of TL3 showed 94% similarity with *Aspergillus aculeatus.* Therefore, the TL3 strain was identified and named *Aspergillus aculeatus,* and the sequence was submitted to NCBI GenBank under accession No. ON387621.

### 3.4. Quantification of Essential Metabolites in Culture Filtrate under Drought Stress

The adaptive pattern of microbial phytohormones and metabolites production upon stress circumstances is one of the methods used by endophytic fungal strains to ensure their survival in abiotic stresses. Under PEG-induced drought stress endophytic fungal isolate WA sufficiently produced higher content of proteins (0.21-fold), sugars (0.15-fold), lipids (0.3-fold), phenols (0.05-fold), flavonoids (1.9-fold) and proline (0.2-fold) compared to the control (-PEG). TG produced higher content of proteins (0.27-fold), sugars (0.14-fold), lipids (0.17-fold), phenols (0.15-fold), flavonoids (0.12-fold) and proline (0.3-fold), under PEG-induced drought stress and TL3 produced proteins (0.2-fold), sugars (0.21-fold, lipids (0.2-fold), phenols (0.05-fold), flavonoids (0.05-fold), and proline (0.9-fold), compared to control (compared to the control (0% PEG) (Figure 2).

### 3.5. Quantification of Hormonal Content in Culture Filtrate under Drought Stress

**Figure 3:**
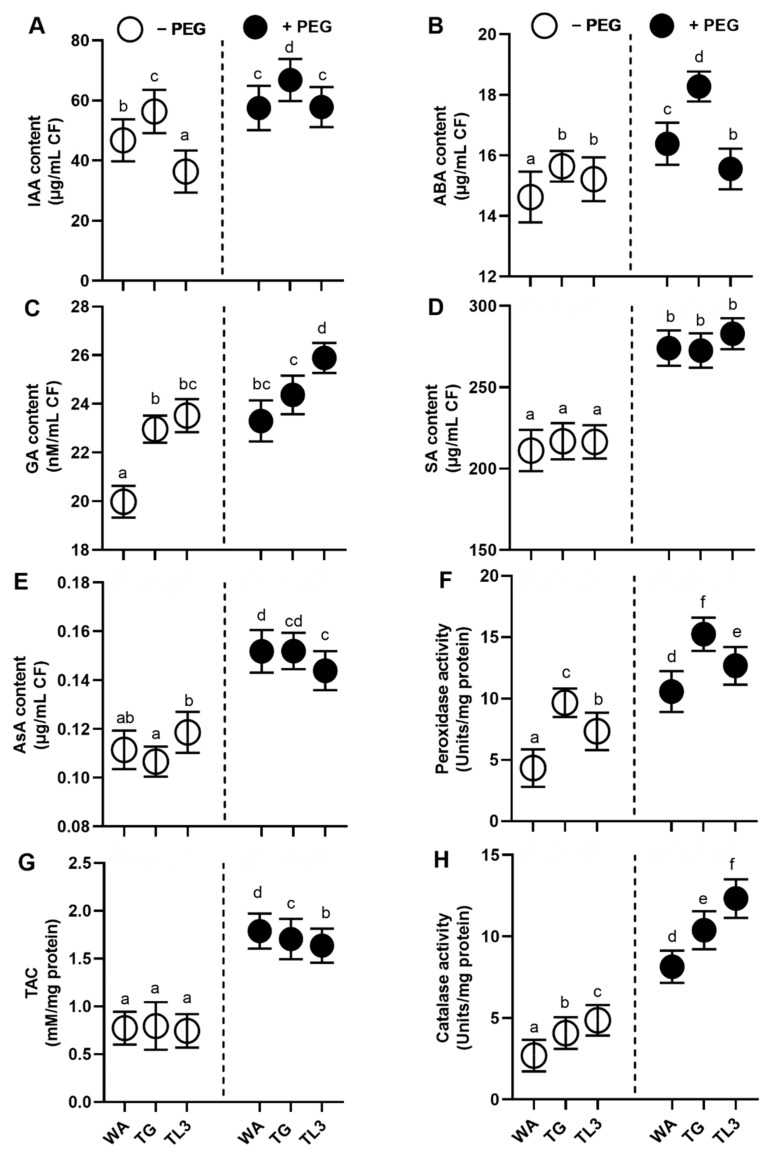
Hormonal contents in CF. (**A**) IAA content, (**B**) ABA content, (**C**) GA content, and (**D**) SA content. Enzymatic and non-enzymatic antioxidants in CF. (**E**) AsA content, (**F**) peroxidase activity, (**G**) total antioxidant capacity, and (**H**) catalase activity. Quantitative data represent means ± SE of three independent biological replicates presented with various letters showing differences at a significance level of *p ≤* 0.05 using Duncan’s multiple range test (DMRT). CF: culture filtrate.

WA, TG, and TL3 isolates were also found to sufficiently produce IAA, ABA, GA, and SA, both under normal and drought stress conditions (Figure 3). Under PEG-mediated drought stress, the level of secreted IAA was sufficiently produced in culture filtrates of WA (0.25-fold), TG (0.23-fold), and TL3 (0.64-fold) compared to control (0% PEG). Under drought stress (8% PEG), the level of secreted abscisic acid (ABA) production in culture filtrate was induced for WA (0.08-fold), TG (0.18-fold), and TL3 (0.06-fold) compared to control (0% PEG). Moreover, the level of secreted GA production in culture filtrate (8% PEG) was also induced for WA (0.21-fold), TG (0.09-fold), and TL3 (0.13-fold) compared to control (0% PEG). SA level in culture filtrates (8% PEG) was induced for WA (0.31-fold), TG (0.19-fold), and TL3 (0.23-fold) compared to control (0% PEG) (Figure 3).

### 3.6. Quantification of H_2_O_2_ and Antioxidants in Culture Filtrate under Drought Stress

WA, TG, and TL3 isolates also ably produced antioxidant enzymes with stronger antioxidant capacity, under PEG-induced drought stress (Figure 3). By supplementing 8% PEG, culture filtrates exhibited significantly (*p ≤* 0.05) higher POX activity by WA (1.5-fold), TG (0.66-fold), TL3 (0.55-fold), CAT activity by WA (3.2-fold), TG (2.3-fold), TL3 (1.75-fold), and total antioxidant capacity by WA (1.8-fold), TG (0.75-fold), TL3 (0.54-fold). The ascorbic acid (non-enzymatic antioxidant) level was also induced by WA (0.6-fold), TG (0.5-fold), TL3 (0.08-fold) in culture filtrate under stress, compared to control (0% PEG) (Figure 3).

### 3.7. PEG-Induced Drought Stress Tolerance Response of M. oleifera Seedlings under In Vitro Conditions

**Figure 4:**
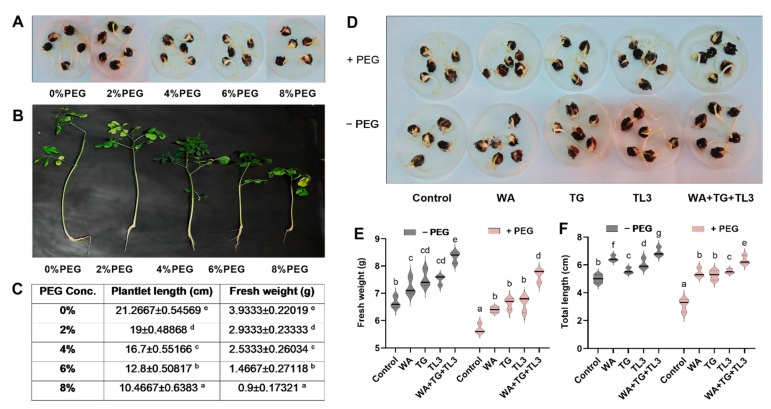
Assessment of PEG-mediated drought stress response of *M. oleifera* seed germination test. (**A**) Germination response in dose-dependent assay under PEG-induced drought conditions (0, 2, 4, 6, 8% PEG), (**B**) phenotypic evaluation of *M. oleifera* plantlets, (**C**) growth assessment of *M. oleifera* germinated plantlets (length and fresh weight), (**D**) seed germination response with endophytic fungal inoculation under control and drought stress, (**E**) seedling fresh weight and, (**F**) seedling length. Quantitative data represent means ± SE of three independent biological replicates presented with various letters showing differences at a significance level of *p ≤* 0.05 using Duncan’s multiple range test (DMRT).

Different concentrations of PEG i.e., 0%, 2%, 4%, 6%, and 8% were applied to *M. oleifera* cotyledons and seedlings, for evaluating the PEG-induced drought stress tolerance ability (Figure 4). Among all concentrations of PEG, 8% PEG proved as intolerant to *M. oleifera* seedlings (7 days old) (Figure 4A). Moreover, the growth response of 15 days old plants under drought stress (8% PEG) was assessed which showed a decline in growth parameters i.e., plantlet length (−0.5-fold) and fresh weight (−0.7-fold) compared to control (0% PEG) (Figure 4B,C). Moreover, the effect of WA, TG, and TL3 individual and combined inoculation of fungal endophytes (with and without PEG) was also assessed based on fresh weight and length from 7 days old seedlings. Results showed that under drought stress (8% PEG), a significant increase was recorded in seedling fresh weight (0.32-fold) and length (1.03-fold) in *M. oleifera* compared to non-inoculated control (8% PEG) (Figure 4D–F).

### 3.8. Effect of Fungal Endophytes on the Growth Attributes of M. oleifera Plants under PEG-Induced Drought Stress under Field Conditions

**Figure 5:**
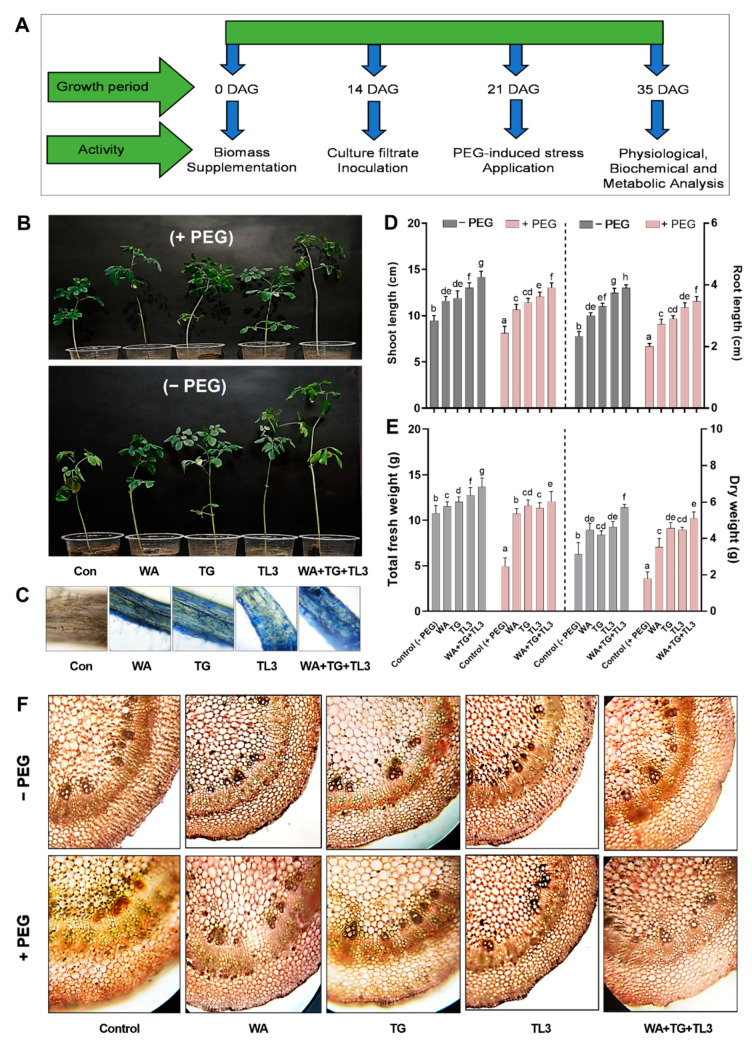
Effect of WA, TG, and TL3 inoculation on *M. oleifera* plant growth. (**A**) Schematic representation of the *M. oleifera* plant bioassay, (**B**) phenotypic analysis; upper penal (plants supplemented with PEG) and lower penal (plants without PEG supplementation), (**C**) root colonization by fungal endophytes, (**D**) shoot length (right penal), and root length (left penal), (**E**) total fresh weight (right penal), and total dry weight (left penal), (**F**) stem anatomical features. Quantitative data represent means ± SE of three independent biological replicates presented with various letters showing differences at a significance level of *p ≤* 0.05 using Duncan’s multiple range test (DMRT).

Under field conditions, the ability of fungal endophytes (WA, TG, and TL3) in the promotion of different growth parameters i.e., seed fresh weight, cotyledon length, shoot, and root length, and fresh and dry weight of *M. oleifera* plants, were recorded at 35 DAS. All fungal isolates individually (with and without PEG-induced drought stress) significantly promoted the growth parameters. However, under drought stress (8% PEG) the combined inoculation of WA, TG, and TL3 predominantly promoted the shoot length (0.73-fold), root length (0.65-fold), shoot fresh weight (1.35-fold), and dry weight (2.13-fold) in *M. oleifera* plants under PEG-induced drought stress, compared to non-inoculated control (8% PEG) (Figure 5). Root colonization by endophytic fungi was also assessed by lactophenol cotton blue staining, which confirmed the successful plant microbe-interaction in the root tissue of *M. oleifera* plants under observations (Figure 5).

### 3.9. Effect of Fungal Endophytes on Stem Anatomical Features in M. oleifera Plants under PEG-Induced Drought Stress

Water deficiency alters the anatomical structure of plant parts. To better understand the effect of fungal endophytes (WA, TG, and TL3) on the drought response of *M. oleifera* anatomy, the stem cross sections were visualized by microscopy. Microscopic observation of stem cross-section showed that the stem sections from *M. oleifera* inoculated with the endophytes showed typical features related to drought tolerance such as water-filled cells, small cell gaps, tight and round cells under normal water conditions, as well as under drought stress conditions. In opposite, stems from *M. oleifera* without endophytic association under drought stress showed slightly deformed mesophyll cells, shorter epidermal and vascular bundle cells, as well as narrower metaxylem, phloem, pith, and cortical area (Figure 5).

### 3.10. Effect of Fungal Endophytes on Photosynthetic Pigments and Growth-Related Metabolites Production in M. oleifera Plants under PEG-Induced Drought Stress

**Figure 6:**
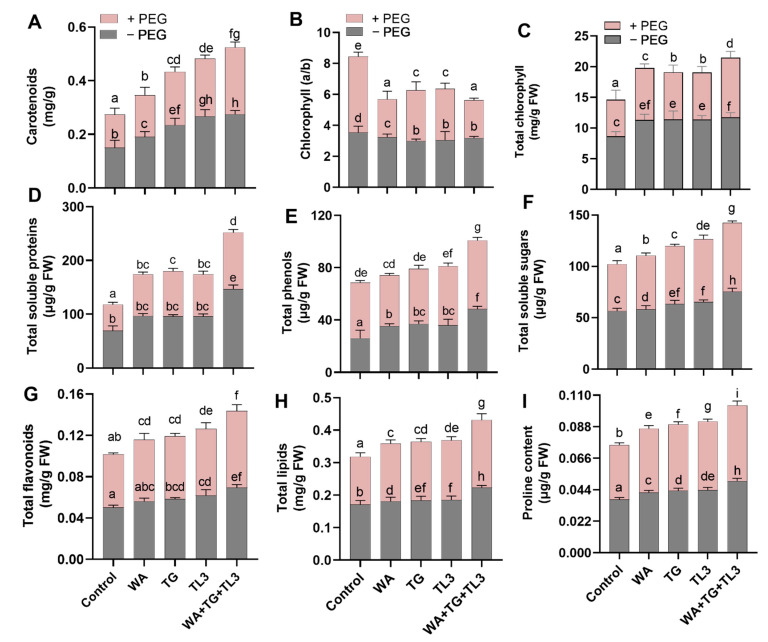
Effect of WA, TG, and TL3 inoculation on *M. oleifera* growth promoting metabolites. (**A**) Carotenoids, (**B**) chlorophyll a/b ratio, (**C**) total chlorophyll, (**D**) total soluble proteins, (**E**) total phenols, (**F**) total soluble sugars, (**G**) total flavonoids, (**H**) total lipids, and (**I**) proline content. Quantitative data represent means ± SE of three independent biological replicates presented with various letters showing differences at the significance level of *p ≤* 0.05 using Duncan’s multiple range test (DMRT).

Similarly, all individual fungal endophytes significantly promoted the chlorophyll and carotenoid contents with and without stress exposure. However, under drought stress (8% PEG), the combined inoculation of WA, TG, and TL3 isolates predominantly promoted the increase in total chlorophyll content (0.46-fold) and carotenoids (0.87-fold) of *M. oleifera* plants compared to the non-inoculated control (8% PEG). While chlorophyll a/b ratio (−0.54-fold) was decreased in *M. oleifera* plants under PEG-induced drought stress compared to the non-inoculated control (8% PEG). There was lesser decrease in chlorophyll a/b ratio in non-inoculated control (8% PEG) (−0.18-fold) compared to the respective control (0% PEG) (Figure 6). Higher values of chlorophyll a/b ratio corresponded to a reduction in photosynthetic activity and initial stress symptoms in plants. Under field conditions, WA, TG, and TL3 isolate individually promoted the production of growth-related metabolites i.e., protein, phenols, sugars, flavonoids, lipids, and proline, of *M. oleifera* (with and without PEG-induced drought stress) at 35 DAS. Basal protein, phenolics, total soluble sugars, lipids, and total flavonoid levels were significantly (*p ≤* 0.05) elevated in response to individual WA, TG, and TL3 isolates compared to non-inoculated control, both under drought stress (8% PEG) and normal conditions (0% PEG). However, in *M. oleifera*, combined inoculation of WA, TG, and TL3 isolates almost doubled the protein level (0.9-fold) compared to the non-inoculated control (0% PEG). In addition to this, *M. oleifera* under drought stress (8% PEG) with combined inoculation of WA, TG, and TL3 isolates significantly (*p ≤* 0.05) prompted maximum protein concentrations (1.2-fold) compared with the non-inoculated control (8% PEG) (Figure 6). The phenolic contents showed a significant (*p ≤* 0.05) increase (up to 0.16-fold) in response to individual WA, TG, and TL3 isolates under drought stress, compared to the non-inoculated control (8% PEG). Moreover, a further increase (*p ≤* 0.05) in phenolic contents (up to 0.66-fold) was noticed in *M. oleifera* with combined inoculation of WA, TG, and TL3 isolates under drought stress compared to the non-inoculated control (8% PEG) (Figure 6). Compared to the non-inoculated control (8% PEG), total soluble sugars, lipids, and proline levels were increased significantly (*p ≤* 0.05) in response to the individual inoculation of WA, TG, and TL3 isolates in maize plants under drought stress. While comparing with the non-inoculated control (8% PEG), a further increase (*p ≤* 0.05) in total soluble sugars (up to 0.42-fold), lipids (up to 0.41-fold), and proline level (up to 0.7-fold) was noticed in *M. oleifera* with combined inoculation of WA, TG, and TL3 isolates (Figure 6).

Compared to the non-inoculated control (8% PEG), a significant (*p ≤* 0.05) increase in flavonoid level (up to 0.6-fold) was also observed in *M. oleifera* with combined inoculation of WA, TG, and TL3 isolates under drought stress (Figure 6).

### 3.11. Effect of Fungal Endophytes on Hormonal Content in M. oleifera Plants under PEG-Induced Drought Stress

**Figure 7:**
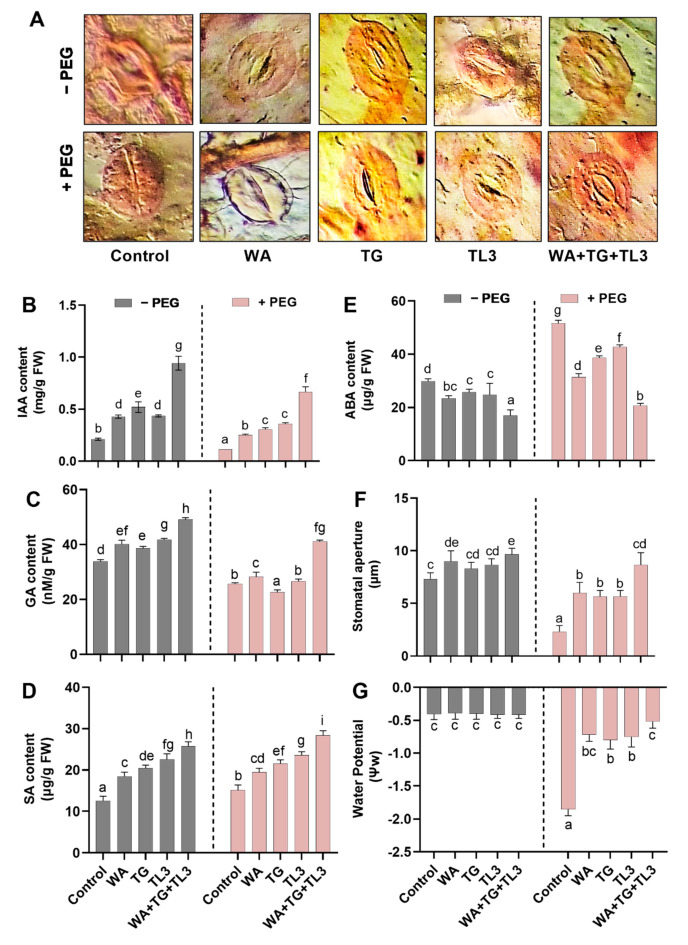
Effect of WA, TG, and TL3 inoculation on *M. oleifera* phytohormonal contents. (**A**) Stomatal anatomy, (**B**) IAA, (**C**) GA, (**D**) SA, (**E**) ABA, (**F**) stomatal aperture, and (**G**) water potential. Quantitative data represent means ± SE of three independent biological replicates presented with various letters showing differences at a significance level of *p ≤* 0.05 using Duncan’s multiple range test (DMRT).

WA, TG, and TL3 isolate individually promoted the production of hormonal content i.e., IAA, GA, SA, ASA, of *M. oleifera* (with and without PEG-induced drought stress), 35 DAS and basal IAA, GA, SA, and ASA levels were also significantly (*p ≤* 0.05) elevated in response to combined inoculation of WA, TG, and TL3 isolates compared with non-inoculated control both under drought stress (8% PEG) and normal conditions (0% PEG). In opposite, basal ABA level was significantly (*p ≤* 0.05) decreased in response to individual and combined inoculation of WA, TG, and TL3 isolates compared with control, both under drought stress (8% PEG) and normal conditions (0% PEG) (Figure 7).

In comparison to the non-inoculated control (8% PEG), *M. oleifera* supplemented with the combined WA, TG, and TL3 isolates, a significant (*p ≤* 0.05) increase of 4.5-fold was noticed in IAA content (Figure 7). In addition to this, *M. oleifera* with combined inoculation of WA, TG, and TL3 isolates under drought stress showed significant (*p ≤* 0.05) elevation in the level of GA and SA (0.7-fold and 0.8-fold, respectively) in comparison to non-inoculated control (8% PEG) (Figure 7).

In the absence of endophytic association, the ABA level was significantly increased (0.79-fold) by drought stress (8% PEG) in *M. oleifera* plants in comparison to the non-inoculated control (0% PEG). Nevertheless, ABA level was significantly (*p ≤* 0.05) reduced in *M. oleifera* by combined inoculation of WA, TG, and TL3 isolates compared with non-inoculated control (8% PEG) as well as non-inoculated control (0% PEG) up to −0.61-fold and −0.31-fold, respectively (Figure 7).

### 3.12. Effect of Fungal Isolates on Stomatal Aperture and Water Potential (Ψw) in M. oleifera Plants under PEG-Induced Drought Stress

The effect of WA, TG, and TL3 isolates was also studied on the stomatal anatomy, stomatal aperture, and water potential (Ψw) of *M. oleifera* under drought stress (Figure 7). The stomatal aperture displayed a significant (*p ≤* 0.05) increase in size under normal (0% PEG) and drought stress conditions (8% PEG) in the individual as well as combined inoculation of WA, TG, and TL3 isolates compared to respective controls.

The maximum increase in stomatal aperture was noticed in *M. oleifera* with combined inoculation of WA, TG, and TL3 isolates under normal (0% PEG) and drought stress conditions (8% PEG), up to 0.45-fold and 3.5-fold, respectively, in comparison to respective controls. While the maximum reduction (−0.75-fold) in stomatal aperture was recorded under drought stress in *M. oleifera* compared to non-inoculated control (0% PEG) (Figure 7).

On the contrary, the maximum decrease in water potential (−3.2-fold) was recorded under drought stress (8% PEG) in *M. oleifera* without inoculation of WA, TG, and TL3 isolates compared to the non-inoculated control (0% PEG). The lesser decreases in water potential were noticed under drought stress in *M. oleifera* with individual inoculation of WA, TG, and TL3 isolates in comparison to non-inoculated control (0% PEG); however, the maximum less decrease (−0.02-fold) in water potential was recorded under drought stress in *M. oleifera* with combined inoculation of WA, TG, and TL3 isolates compared with the non-inoculated control (0% PEG) (Figure 7).

### 3.13. Effect of Fungal Endophytes on Antioxidant System in M. oleifera Plants under PEG-Induced Drought Stress

**Figure 8:**
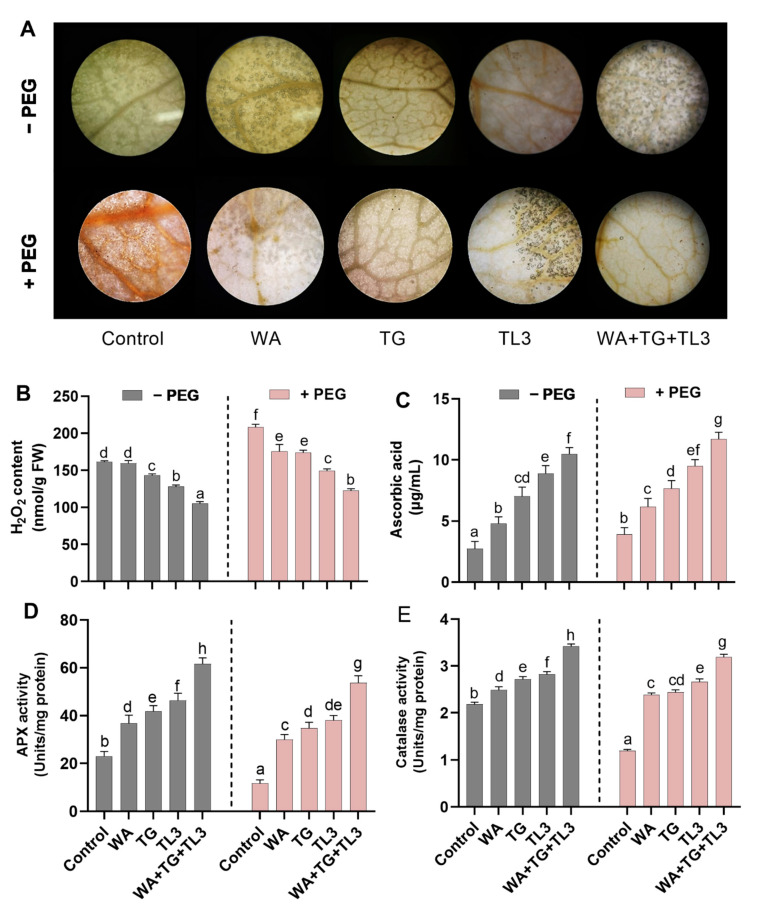
Effect of WA, TG, and TL3 inoculation on *M. oleifera* ROS production and antioxidant system. (**A**) DAB staining using leaf segment from 35-d-old plants, (**B**) H_2_O_2_ level, (**C**) AsA, (**D**) ascorbate peroxidase activity, and (**E**) catalase activity. Quantitative data represent means ± SE of three independent biological replicates presented with various letters showing differences at a significance level of *p ≤* 0.05 using Duncan’s multiple range test (DMRT).

In response to drought stress, oxidative damage in terms of ROS production was studied in *M. oleifera* upon inoculation of WA, TG, and TL3 isolates. To this end, the amount of H_2_O_2_ was observed as brown spots by using DAB (3,3ʹ-diaminobenzidine) staining in the leaves of *M. oleifera* (Figure 8). The highest amount of H_2_O_2_ accumulation was recorded in PEG-treated plant tissues, up to 0.29-fold in comparison to non-inoculated control (0% PEG). While, decreased H_2_O_2_ accumulation (−0.4-fold) and DAB staining was observed in *M. oleifera* with combined inoculation (WA, TG, TL3) under drought stress in comparison to non-inoculated control (8% PEG) (Figure 8).

The antioxidants (enzymatic and non-enzymatic) were studied in *M. oleifera* with individual and combined inoculation (WA, TG, TL3) under drought stress. Differential responses observed under different treatments are shown in Figure 8. The ASA (non-enzymatic antioxidant) level was also significantly (*p ≤* 0.05) increased under normal and drought stress conditions upon the individual as well as combined inoculation (WA, TG, TL3) compared to control (0% PEG). The ASA content was significantly increased under normal (1.68-fold) and drought stress condition (1.85-fold) in *M. oleifera* with combined inoculation (WA, TG, TL3) in comparison to the respective controls, 0% PEG and 8% PEG, respectively (Figure 8).

Similarly, the activity of the peroxidase enzyme displayed a significantly (*p ≤* 0.05) higher increase under normal and drought stress conditions upon individual inoculation of WA, TG, and TL3 isolates compared to the respective control. However, the peroxidase (POX) enzymatic activity was further increased (*p ≤* 0.05) under normal (1.36-fold) and drought stress (3.5-fold) in *M. oleifera* with combined inoculation (WA, TG, TL3) in comparison to the respective controls, 0% PEG and 8% PEG, respectively (Figure 8). Like peroxidase, the catalase activity was also augmented in *M. oleifera* with individual and combined inoculation (WA, TG, TL3) under drought stress. The catalase (CAT) enzymatic activity was further significantly (*p ≤* 0.05) increased in *M. oleifera* with individual and combined inoculation (WA, TG, TL3) under normal (0.6-fold) and drought stress condition (1.7-fold) in comparison to the respective controls, 0% PEG and 8% PEG, respectively (Figure 8).

### 3.14. Multivariant Assessment by Principal Component Analysis (PCA)

To finalize the cumulative evaluation of all the variables under the current study, we have taken advantage of a suitable statistical method; principal components analysis (PCA) ideal for evaluating and revealing associations between endophytes inoculation and plant traits under drought stress (Tabachnick and Fidell, 2007), as it comprehensively lowers many observable variables to a smaller number of elements.

**Figure 9:**
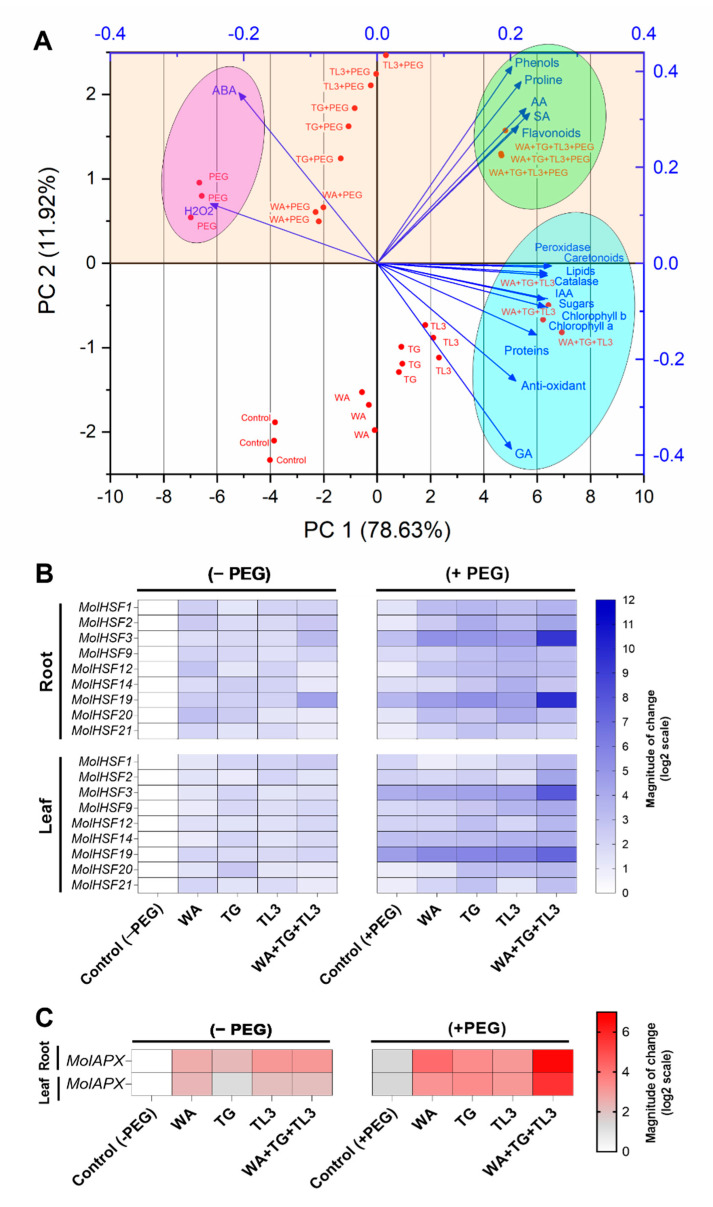
(**A**) Principal component loading plot and scores of principal component analysis (PCA) of physiochemical parameters, chlorophyll b, chlorophyll a, carotenoids, proteins, phenols, sugars, flavonoids, lipids, proline, IAA, GA, SA, AA, peroxidase, anti-oxidant, catalase, ABA and H_2_O_2_ of *M. oleifera* as affected by PEG-mediated drought stress under different endophytic treatments: WA, TG, and TL3 alone or in combination (WA, TG, and TL3) compared with the non-inoculated respective controls (0% PEG and 8% PEG). The scores show the contribution of individual treatments on plant traits, as indicated by the direction and magnitude of the eigenvector vectors, (**B**) expression profiling of *HSF* genes, and (**C**) *APX* gene measure by RT-qPCR. Quantitative data represent the means ± SD of three independent experiments and at least three technical replicates each.

An overview of *M. oleifera* response upon PEG-mediated drought stress with the different endophytic inoculations (WA, TG, TL3) alone or in combination, and findings of principal component analysis are shown in Figure 9. The first two principal components (PCs) were related, with eigenvalues higher than 2, and explained the total variance, with PC1 and PC2 accounting for 78.63% and 11.92% respectively (Figure 9 and Supplementary Table S4). PC1 was positively correlated to chlorophyll b, chlorophyll a, carotenoids, proteins, phenols, sugars, flavonoids, lipids, proline, IAA, GA, SA, AA, peroxidase, antioxidant, and catalase. PC1 was also negatively correlated to ABA and H_2_O_2_. Moreover, PC2 was positively correlated to proline, flavonoids, and phenols, ABA, SA, AA, and H_2_O_2_; while PC2 was negatively correlated to chlorophyll b, chlorophyll a, carotenoids, proteins, sugars, lipids, IAA, GA, peroxidase, antioxidant, and catalase (Figure 9 and Supplementary Table S5).

The treatments were well separated and distributed as shown in the loading plot (Figure 9A and Supplementary Table S6). The non-inoculated control (0% PEG) was positioned on the negative side of PC1 in the middle of the lower-left quadrant. The endophytic inoculations (WA, TG, TL3) alone were positioned on the negative side of PC1 in the lower left quadrant for WA, and the lower right quadrant for TG, and TL3 inoculations. While the endophytic inoculations (WA, TG, TL3) in combination were positioned on the positive side of PC1 in the lower right quadrant (Figure 9). Treatments with PEG-mediated drought stress (8% PEG) without endophytic inoculation and with individual alone endophytic inoculations (WA, TG, TL3) were positioned on the negative side of PC1 in the left upper quadrant. While the treatments with PEG-mediated drought and endophytic inoculation (WA, TG, TL3) in combination were positioned on the negative side of PC1 in the right upper quadrant (Figure 9).

The distribution of the treatments and plant traits across the measured factors in PC1 was strongly attributed to the differential physio-hormonal and biochemical responses under drought stress upon endophytic co-inoculation (WA, TG, TL3) in comparison to control (8% PEG) as well as non-inoculated control (0% PEG) (Figure 9). The two axes represent 78.63% and 11.92% of the data variance and PCA scores showed a significant correlation of endophytic fungal treatment under drought stress to the separation of the physio-hormonal and biochemical traits as indicated by the direction and magnitude of the respective vectors (Figure 9).

### 3.15. Quantitative Gene Expression Analysis of MolHSFs and MolAPX under Drought Stress

The RT-qPCR analysis was carried out to quantify the expression level of selected *MolHSFs* and *MolAPX* in the leaf and root tissues of *M. oliefera* grown under drought stress. The analysis revealed that among selected *HSF* genes, *MolHSF3* exhibited a significantly higher expression in the root (2.1-fold) as well as leaf (1-fold) tissue of *M. oliefera* grown under drought stress, in response to the co-inoculation of WA, TG, and TL3, compared with the non-inoculated control (8% PEG). *MolHSF19* also showed increased expression (>5-fold) in the root (0.7-fold) as well as leaf (1.1-fold) tissue of *M. oliefera* grown under drought stress, in response to the co-inoculation of WA, TG, and TL3, compared with the non-inoculated control (8% PEG) (Figure 9). *MolAPX* also exhibited significantly increased expression in the root (>3.7-fold) and leaf (>3-fold) tissue of *M. oliefera* grown under drought stress, in response to the co-inoculation of WA, TG, and TL3, compared with the non-inoculated control (8% PEG) (Figure 9).

**Figure 10:**
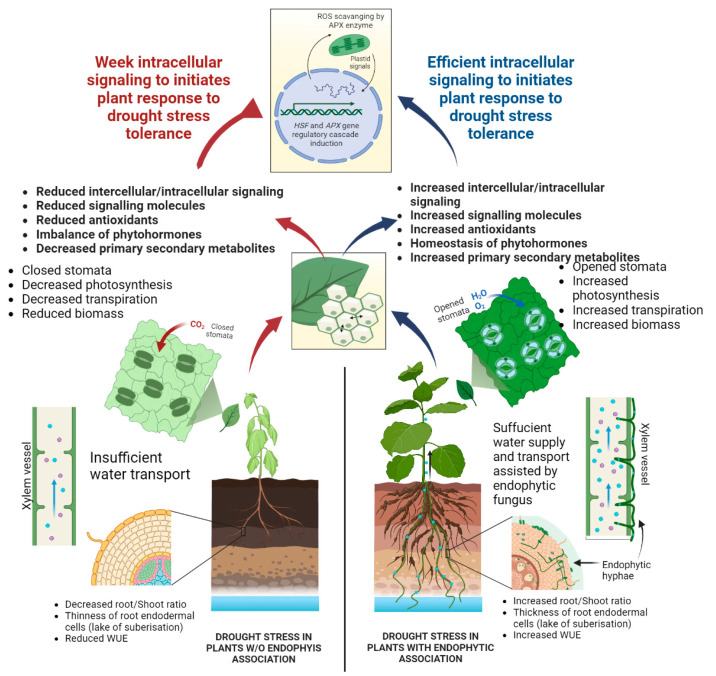
Working model showing a molecular mechanism regulating the drought stress tolerance in *M. oleifera* driven by *MolHSF3* and *MolHSF19* transcription factors and *APX* genes induced by growth-promoting, antioxidant-rich, endophytic fungal consortium (WA, TG, and TL3).

Overall, the working model has depicted a molecular mechanism regulating the drought stress tolerance in *M. oleifera* driven by *MolHSF3* and *MolHSF19* transcription factor genes that are induced by growth-promoting, antioxidant-rich, endophytic fungal consortium (WA, TG, and TL3). *MolHSF3* and *MolHSF19* induced the expression of the antioxidant enzymatic gene *(APX),* downstream to *MolHSF3* and *MolHSF19* regulatory cascade. APX enzyme finally initiated the ROS scavenging leading to drought stress tolerance in *M. oleifera* plants (Figure 10).

## 4. Discussion

The findings of the present investigation suggested that the beneficial, growth-promoting endophytic fungal consortia efficiently enhanced the basal drought tolerance in *Moringa oleifera* by inducing antioxidant enzymes specifically *APX* through induction of heat shock factor genes.

The current research was rationalized in the scenario of the fact that global climate change is resurfacing, with major increases in temperature resulting in abiotic stresses such as drought stress, which harm agricultural and forestry yield. In such a diverse range of adverse conditions, supportive approaches and the use of environmentally friendly technologies can help break the feedforward loop by improving resource use efficiency and expanding yield [46], to improve healthy crop growth and reforestation while refraining from unsustainable efforts, thus monitoring risky environmental conditions, and to improve soil health by sequestering soil carbon, sustaining soil fertility, and even to enhance the soil quality by sequestering soil carbon, upholding soil fertility [47]. Due to drastic climate change, the growth and productivity of some important plants including *M. oleifera* significantly showed a negative growth impact. However, scientists applied different approaches including synthetic chemicals, nutritional (plant/seaweed extracts), and biological amendments (endophytic and rhizosphere microbes) to ameliorate stresses [48,49]. The application of such approaches is of specific interest in the conception of seedling’s response under drought stress and to gain knowledge for the success of reforestation programs based on woody species such as *Pinus Halepensis* [50], and *M. oleifera.* Among these approaches, the use of microbes, especially endophytic fungi are of key importance in different stresses alleviations, such as salinity, heavy metal, and waterlogging stresses by conferring the agronomic and adaptive benefits to the host plants under several abiotic stresses such as tenacious drought, adversative temperatures, soil salinity, waterlogging, and heavy metal toxicity [51,52].

The exploitation of beneficial microbes is the most realistic, reliable, and maintainable approach for mitigating the problems of abiotic stress and their influence on plant growth, productivity, and yield [26,53]. Recognition of drought-tolerant endophytic fungi linked with flora growing in harsh conditions, as well as their range, communal structure, and environmental variables impacting them, is thus of substantial biotechnological significance.

Drought stress, in the arid zone, implicates more severe damage to plants including *M. oleifera* which thrives in tropical, subtropical, and temperate regions and is a member of the *Moringaceae* family well-known for its medicinal values. It has previously shown satisfactory growth and development in an open field of the arid zone under moderate drought stress. But to what extent it bears drought stress and whether can it be mitigated by various strategies, must be reported yet. Moreover, the detailed morphophysiological, biochemical, and molecular mechanism for drought tolerance in *M. oleifera* is not well characterized so far.

Keeping in mind the previous knowledge, the current investigation was rationalized to unravel the role of endophytic fungi *Microdochium majus* (WA), *Meyerozyma guilliermondi* (TG), *Aspergillus aculeatus* (TL3) isolated from drought adapted *Carthamus oxyacantha* L. for investigating their symbiotic potential on *M. oleifera* under drought stress. The level of endogenous phytohormones changes because of environmental perturbation, which modifies plant development for better survival. Unfortunately, not all plant species can rebalance the endogenous hormonal contents. Therefore, endophytes producing growth-promoting hormones and metabolites have always been considered as best suitable for plant growth promotion. IAA is one of the most important phytohormones in plants since it regulates their growth and development. The current study showed the strong capability of WA, TG, and TL3 isolate to produce IAA, proline, total phenols, flavonoid, proteins, and lipids indicating its potential role in the induction of growth responses in plant system. Interestingly, IAA, proline, total phenols, flavonoids, proteins, lipids, and antioxidants were significantly produced upon PEG-mediated drought stress imposed on WA, TG, and TL3 endophytes, rendering them suitable as drought stress ameliorating endophytes for plants.

There is no previous report so far that could introduce the species of *Microdochium* *majus*, *Meyerozyma guilliermondi,* and *Aspergillus aculeatus* as osmotic or water stress tolerant microorganisms. The current report is the first one to explore the drought stress-tolerant behavior of *Microdochium* *majus*, *Meyerozyma guilliermondi,* and *Aspergillus aculeatus* isolates that proved to bear significant PEG-mediated drought stress tolerance by producing enough biomass and growth-promoting metabolites. Recently, [54] reported that under dry conditions, inoculation of endophytic strain *Phoma *sp. boosted seedling development of *Pinus tabulaeformis.* Moreover, only a few previous reports are showing the induction of drought stress tolerance by endophytes in plants, such as *Epichloë* endophyte induced the drought tolerance in *Festuca arundinacea* and *Lolium perenne* [55], consortia of *Penicillium Phaeosphaeria*, *Penicillium chrysogenum, Penicillium brevicompactum*, *Alternaria* sp., and *Eupenicillium osmophilum* induced drought resistance in *Colobanthus quitensis* [56], the combination of fungal isolates SMCD 2206, 2210, and 2215 belonging to *Ascomycota* drought tolerance in wheat [57], an endophytic consortium induced drought tolerance in hybrid popular [58], arbuscular mycorrhiza protected the chicory (*Cichorium intybus* L.) against drought stress [59], and *Cinnamomum Migao* seedlings [60]. In addition, [61] found that an endophytic fungus (*Talaromyces omanensis)* enhanced the multiplicative, physiochemical, and anatomical traits of drought-stressed tomato plants. 

Polyethylene glycol (PEG) is a long-chain polymer present in a different range of molecular weights which induced drought stress in different plant species. Researchers have used it to assess the drought resistance of crops during seedling establishment since it inhibits seed germination and development by lowering water potential, and the effect is more noticeable on shoots than on main roots. In vitro screening with PEG has been shown in several studies to be one of the most accurate procedures for selecting drought-tolerant genotypes based on germination indices [62].

Many metabolic functions, such as photosynthesis, avert carbon uptake, triggering injury to the photosynthetic machinery (Razi and Muneer, 2021). Previous research has shown that stomatal restraint under mild to moderate drought stresses and non-stomatal limitation under severe drought conditions induced a reduction in leaf photosynthesis [63], as both Chl a and Chl b are known to decrease under drought stress, although the chlorophyll a/b ratio tends to increase due to a higher decline in Chl b than in Chl a. Plants in every environment have more Chl a than Chl b. It is critical to keep more Chl a than Chl b to survive. As a result, Chl b may be changed to Chl a during the Chl degradation process, increasing Chl a content. Thus, the increased chlorophyll a/b ratio shows the reduction in photosynthetic activity and initial stress symptoms in plants.

Present findings consistently showed an increase in total chlorophyll contents (chlorophyll a, chlorophyll b, and carotenoids) and reduced chlorophyll a/b ratio of *M. oleifera* inoculated with fungal endophytes (WA, TG, TL3) under drought stress (8% PEG), which is attributed to enhanced photosynthesis and accelerated growth attributes for biomass production (shoot and root length, fresh and dry weight) of *M. oleifera* plants under stress.

Current results also exhibited higher levels of phenolics, flavonoids, and H_2_O_2_ might be due to the degradation of macromolecules within plants under drought stress. Likewise, higher proline content could be due to the accelerated protein degradation, and/or it is produced as a signal for osmoprotectant activity in PEG-treated *M. oleifera* under stress, along with the reduced quantity of total proteins, lipids, and sugar content. Previously it is also known that plants under drought stress exhibit excess accumulation of ROS that damages DNA, lipids, proteins, and carbohydrates, and ultimately result in irreversible damage and cell death [64].

In present study the plants with endophytic inoculation (WA, TG, TL3) showed a reversal of this behavior with a higher protein, lipid, sugar, and reduced H_2_O_2_. While, the proline (osmoprotectant), phenolics, and flavonoids were further higher in *M. oleifera* plants inoculated with endophytes, under PEG-stress suggestive of an optimal osmoprotectant and antioxidant activity of secondary metabolites inducing stress tolerance in *M. oleifera.*

Consistent with the previous reports, the current study is also suggestive of the osmoprotective role of proline produced in *M. oleifera* inoculated with endophytes (WA, TG, TL3) under PEG-induced drought stress. Environmental stressors such as high temperatures, drought, and waterlogging are also known to limit crop development and overproduction of proline, indicating metabolic re-adjustments for stress tolerance in plants [65]. Previously, the foliar application of proline enhanced the internal free proline in plants thereby increasing drought tolerance [66].

Drought stress causes an increase in ROS production, which must be regulated in a homeostatic pool. Plant ROS are chemically reactive oxygen metabolites and their derivatives, mainly including hydrogen peroxide (H_2_O_2_), superoxide anions (O^2−^), hydroxyl radicals (HO^−^), and singlet oxygen (_1_O^2^). High levels of ROS produce oxidative stress, causes protein denaturation, lipid peroxidation (by producing MDA; a product of lipid peroxidation in biomembranes degradation), nucleotide disruption, and may alter plant physiology, ultimately leading to plant death [11,67]. At a certain level, ROS works as an indicator molecule for activating acclimatory/protection responses through transduction pathways, where H_2_O_2_ acts as a secondary messenger. However, additional ROS induces harmful effects on plant cells. As a result, defenses against ROS are activated by an array of nonenzymatic antioxidants [metabolites such as ascorbate (AsA), carotenoids, glutathione (GSH), and proline] and antioxidant enzymes [such as guaiacol peroxidases (GPOX), catalase (CAT), superoxide dismutase (SOD) and AsA-GSH cycle enzymes like glutathione reductase (GR) ascorbate peroxidase (APX), monodehydroascorbate reductase (MDHAR), dehydroascorbate reductase (DHAR)], work together for detoxification of ROS under drought stress [68,69,70,71].

Ref. [72] also observed that endophyte inoculations hindered ROS accumulation in the plant cell by activating antioxidant enzymes. Current findings also exposed that APX gene expression and ROS scavenging enzymes (APX) activity was induced in *M. oleifera* inoculated with endophytic fungi under drought stress. This scenario could be possible due to dual signaling mechanisms active for drought resistance in plants: (1) Due to cell signaling initiated by signaling molecules produced within plants by plant cells; (2) due to the signaling molecules produced and secreted by antioxidant-rich fungal mass residing within plant tissues (roots, stem, and leaf). Total antioxidant capacity was also increased by combined inoculation (WA, TG, TL3), thereby overcoming drought induced oxidative stress in *M. oleifera.* Previous reports have indicated that ROS is involved in plant response to drought stress through regulating ROS scavenging systems. *SNAC3* transcription factor in rice was induced by drought and reduced ROS damage by activating the expression of ROS scavenging enzyme genes [73]. The drought inducible *Hsfs* are also known to control the expression of the *APX* gene by interacting with the *Hsf*-binding motifs in the promoter of *APX* involved in the ROS gene network [74].

Several reports have shown that fungal endophytes can provide the plant with long-lasting resistance to biotic and abiotic stresses, by balancing the various phytohormone-dependent pathways, that modulate the levels of growth and defense regulatory proteins under normal and stressed conditions [26]. Our results also showed that endophytic combined inoculation (WA, TG, TL3) not only produced and provided but also induced the production and accumulation of IAA, GA, and SA levels in *M. oleifera* under drought stress. IAA has previously been shown to promote abiotic stress tolerance by inducing antioxidant enzyme activity, gene expression, photosynthetic pigment accretion, and osmoprotectant production, such as proline.

Previously, SA is known for abiotic stress signaling in a variety of physiological processes including stomatal closure, thermogenesis, metabolism, osmolyte, photosynthesis, blooming, fruit quality enhancement, water balances, and antioxidant defense systems [75]. Salicylic acid treatment increased wheat catalase activity [75]. Consistently, our results also revealed that fungal endophytes not only sufficiently produced SA upon PEG-induced drought stress but also induced the basal level of SA in *M. oleifera* under drought stress. That could be one of the reasons for drought stress amelioration by individual inoculation of WA, TG, and TL3 isolates. Moreover, this response was further amplified by combined inoculation (WA, TG, TL3) in plants under drought stress, and SA production was also exponentiated, likewise further contributing to the drought tolerance response.

Gibberellins (GAs) are another important plant growth hormone that contributes to increased plant antioxidant activities for ROS scavenging and photosynthetic properties (stomatal conductance, net photosynthesis, photosynthetic oxygen evolution, and carboxylation efficiency), cell cycle regulation (the division and expansion of cell growth, radicle cell development in a meristem), and germination rate in stressed plants [76]. Our results also revealed that WA, TG, and TL3 isolates sufficiently produced GA upon PEG-mediated drought stress and induced the basal GA level in *M. oleifera* as well, which sufficiently co-operated with other metabolites for sustainable development of *M. oleifera* under drought stress.

Previously, water stress generated a rapid and robust stomatal closure, driven by abscisic acid (ABA) buildup, resulting in photosynthesis inhibition and detrimental impacts on *M. oleifera* biomass production [77]. Current findings consistently suggested that overproduction of ABA in *M. oleifera* grown under drought stress is involved in prolonged stomatal closure leading to photosynthesis inhibition, low sugar synthesis, and less biomass production. Inoculation of WA, TG, and TL3 isolates not only supplied ABA but also induced adequate ABA production with moderately activated stomatal closure, resulting in sufficient photosynthetic activity, and soluble sugar biosynthesis, as well as biomass production in *M. oleifera* under stress.

By taking advantage of PCA approach, the statistical assessment of the influence of endophytic fungal strains (WA, TG, TL3) on *M. oleifera* under drought stress and normal conditions was evaluated revealing differential responses of *M. oleifera* driven by endophytic fungi under normal and drought stress condition. We consider that the first two main components provided enough information to support the main results presented in the current study. As reported in previous studies, the production of antioxidants, ROS, proline, ABA, phenolics, and flavonoids are reliable indicators of drought stress tolerance in plants. This study has also provided evidence that ABA and H_2_O_2_ rebalancing is highly correlated with several biochemical and physiological parameters including proline, photosynthetic pigments, peroxidase, catalase, ascorbate, phenols, flavonoids, carotenoids, lipids, sugars, proteins, and phytohormones (GA, IAA, and SA), which could be used as supplementary indicators for drought stress tolerance factors. Biplot drawn at the control and drought stress conditions showed the association between *M. oleifer* and endophytes’ growth traits under control (0% PEG) and drought stress conditions (8% PEG), which have projected diametrically extreme opposite ends of the biplots compared with the non-inoculated plants.

Moreover, plants have evolved a variety of molecular mechanisms in response to drought stresses which affect the growth and development of plants. For exploring and elucidating the molecular mechanisms of stress resistance, the higher quality of the genome assemblies of significant plants has been prioritized by scientists for extracting the valued genomic entities, as, genomes and transcriptomics can provide vital resources to aid crop modification. [21] have recently performed genome assembly of *M. oleifera* var. Bhagya uses long reads (PacBio) and short reads (Illumina) to generate better genome coverage. The phylogenetic study indicated that *M. oleifera* has *WRKY*, *zinc finger*, as well as *HSFs* known for their role in abiotic stress responses [78]. Despite their name, *HSFs* also influence response to other stresses like as cold, salt, and drought, in addition to heat stress [22]. Recently, phenotypic, and physiological assays showed that overexpression of *OsHSFA3* in *Arabidopsis* conferred drought tolerance by reducing water loss and reactive oxygen species (ROS) levels, whereas it increased abscisic acid (ABA) levels and expression of *AtADC1, AtADC2, SPDS1,* and *SPMS* genes related to polyamines biosynthesis [79]. Another report has shown that in rice, expression levels of HSF genes *OsHsfA4a* and *OsHsfA2* were upregulated under ABA and ROS treatments and thus regulate stress tolerance [80]. [21] discovered the expression of *MolHSFs* in *M. oleifera* plants exposed to drought stress at the early stages of growth. In the genome of *M. oleifera*, 21 putative *HSFs* have been identified and classified according to the HR-A/B domain responsible for protein interaction activity of HSFs. This conserved behavior of TFs throughout evolution renders *HSFs* a crucial part of *M. oleifera* plants’ growth and survival along with development. HSFs have been known to act as molecular sensors to directly sense ROS signals, which in turn activate the expression of HSPs and oxidative stress response genes. Previously, it has been reported that the expression of Hsfs triggers changes in the expression of downstream target genes. One of the major targets of *Hsfs* is the *APX* and Hsf-binding motifs have been detected in the promoter region of genes involved in the ROS gene network, including defense genes, H_2_O_2_-scavenging enzyme APX, and related transcription factors [75].

Here, we specifically investigated the expression levels of these *HSF* and *APX* (direct target of HSF) regulatory cascade genes under drought stress, and consistent with the previous findings and reports, our study also revealed the basal expression of *MolHSFs* and *MolAPX* in leaf and root tissues. While most *MolHSFs* were found predominantly upregulated in root tissue compared to leaf tissue, as well as *MolAPX*. This confirms previous findings that the root is the primary tissue exposed and responds to drought stress, quicker than leaves, experiencing more multifaceted gene regulation upon water deprivation. However, *MolHSF3* and *MolHSF19* presented the highest expression among all the selected *MolHSFs* of *M. oliefera*, suggestive of a critical role in regulating drought stress tolerance due to a significant and ultimate increase in the expression of *APX* gene in *M. oleifera* plants inoculated with fungal endophytes (WA, TG, TL3) upon drought stress triggering the ROS scavenging and signaling.

## 5. Conclusions

The present investigation is concluded with an emphasis on the potential role of WA, TG, and TL3 endophytic fungi in the growth promotion of *M. oleifera* by sufficiently producing growth-promoting hormones, primary and secondary metabolites. Moreover, drought stress tolerance was also ameliorated by endophytic fungal inoculation (WA, TG, TL3) in *M. oleifera* grown under stress. Stress tolerance was enhanced by the production of proline, SA, phenolics, flavonoids, enzymatic antioxidants (APX and CAT), and nonenzymatic antioxidants (ascorbic acid) in *M. oleifera.* The current study is suggestive of using WA, TG, and TL3 endophytic fungi as bio stimulators with strong antioxidative potential for activating plant growth and alleviating the drought stress, especially in arid regions, under dry and xeric environments. PCA and molecular analysis in *M. oleifera* have also exposed the fungal-plant communication and signaling mechanisms that revealed the *MolHSF3*, *MolHSF19,* and *MolAPX* gene regulatory cascade for drought stress tolerance by elevating the antioxidant potential of *M. oleifera* under drought stress.

Hence, current research is referring to the present findings as useful not only for the scientific communities of the pharmacological and pharmaceutical industry but also for the farmers and likewise for the forest scientists in the agroforestry industry to utilize this newly reported endophytic consortium as a bioengineer and bio-stimulant for the alleviation of drought stress in *M. oleifera* to promote the sustainable agroforestry in drought-prone regions throughout the world due to drastic climate change.

## Data Availability

The whole datasets presented in this article have been included in the manuscript and submitted to NCBI repository. Requests for further information should be directed to M.R., mamoona@awkum.edu.pk.

## Supplementary Materials

The following supporting information can be downloaded at: https://www.mdpi.com/article/10.3390/antiox11091669/s1, Figure S1: Molecular identification of fungal endophytes (A) WA, (B) TG, and (C) TL3M9, showing phylogenetic trees constructed with bootstrap replications of 1 K; Table S1: Experimental setup with treatments; Table S2: Endophytic Fungal Strains Isolated from *Carthamus oxyacantha* L.; Table S3. List of primers used for qRT PCR; Table S4: Showing eigenvalues and percentage of variance with cumulative percentages; Table S5: Showing eigenvector magnitudes in terms of coefficients of PC1 and PC2; Table S6: Sowing PCA score data for various treatments for PC1 and PC2.
