# Supplementary material for: Endophytic Fungal Consortia Enhance Basal Drought-Tolerance in Moringa oleifera by Upregulating the Antioxidant Enzyme (APX) through Heat Shock Factors
Source: Antioxidants (Basel). 2022 Aug 27;11(9):1669. doi: 10.3390/antiox11091669 (PMC9495891; doi:10.3390/antiox11091669)
Supplement: Supplementary file 1 [file antioxidants-11-01669-s001.zip › antioxidants-1860315-supplementary.pdf]

Supplementary Figure legends

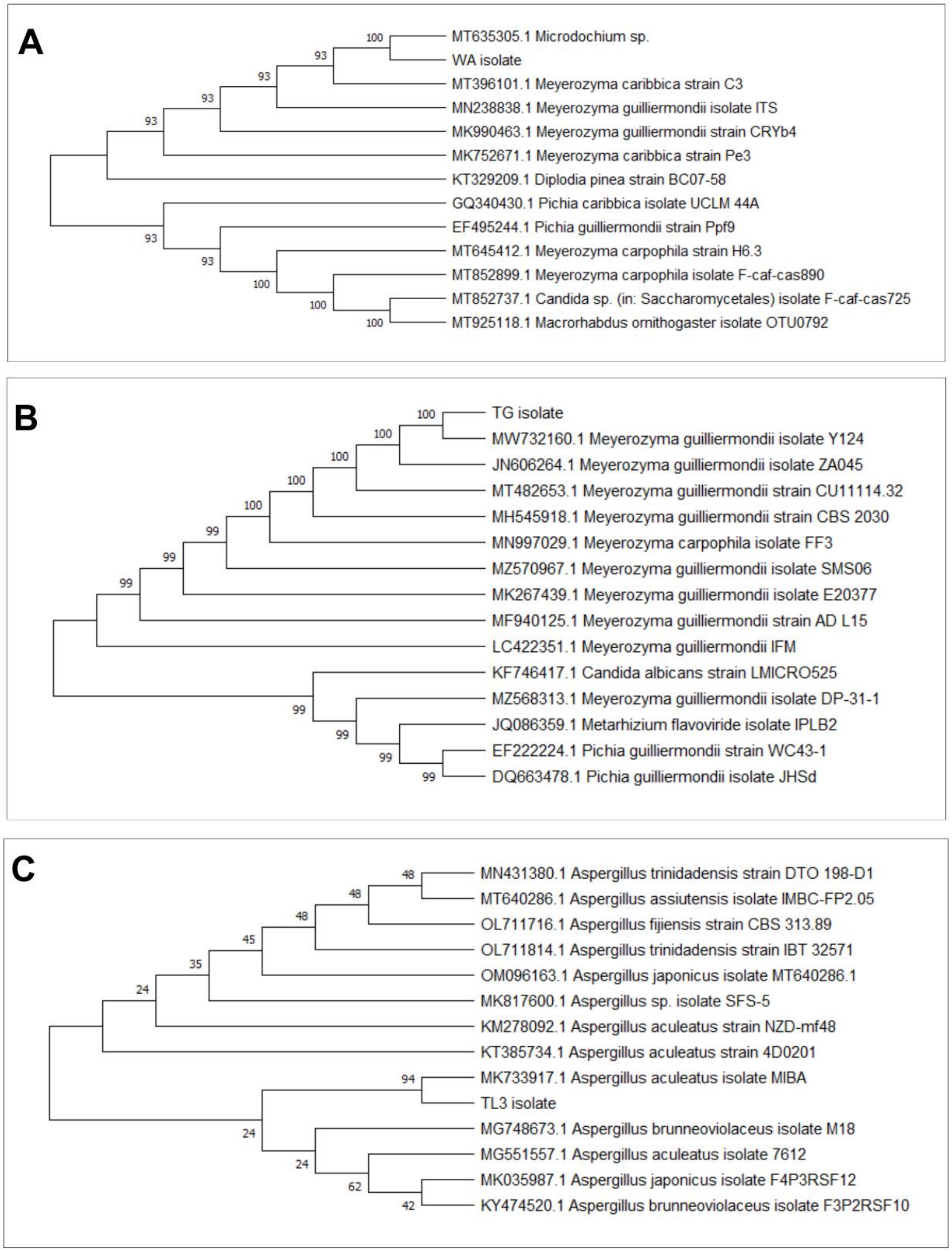

Supplementary Figure S1. Molecular identification of fungal endophytes (A) WA, (B) TG, and (C) TL3M9, showing phylogenetic trees constructed with bootstrap replications of 1 K.

Supplementary Table legends

Supplementary table S1. Experimental setup with treatments.

Treatment (-PEG)	Treatment (+PEG)
Control (+ irrigation water)	Control (+8% PEG)
Plants treated with WA	Plants treated with WA + 8 % PEG
Plants treated with TG	Plants treated with TG + 8 % PEG
Plants treated with TL3	Plants treated with TL3 + 8 % PEG
Plants treated with WA + TG + TL3	Plants treated with WA + TG + TL3 + 8 % PEG

Supplementary table S2. List of primers used for qRT-PCR

GENE	FORWARD PRIMER	REVERSE PRIMER
HSF1	*CCCAGGAGCCAATAAGAGCC*	*GGACCCCAAAGAGCTTCACA*
HSF3	*ACTGGAACATGCCCACGAAA*	*GCAATGGCGAAGATGCCAAA*
HSF12	*ACCCTCCATCTCTGCTCCTT*	*AATGCACCAGACCCTGTCTC*
HSF19	*AAGTAGGAGTGGGGAGGGAG*	*GCAAGTGCTTCCAGCTCTGA*
HSF21	*AGTCAACAACCCTGGCACTT*	*GGATGTGCCACTGACAGGAA*
APX	*TCGAGCCGATCAAGGAGCAG*	*GCAAAGAAAGCATCCTCATC*
ACTIN	*TGGAAAGTGTCAAAGTGGGG*	*CGATAATAACAACAGTAATGGCA*

Supplementary table S3. Endophytic Fungal Strains Isolated from *Carthamus oxyacantha* L.

Host plant	Portion of plant	Segments per plate	No. of emerged Endophytic fungi
Carthamus oxyacantha	Leaf	12	05
	Stem	12	05
	Root	12	05

Supplementary table S4. Showing eigenvalues and percentage of variance with cumulative percentages.

No.	Eigenvalue	Percentage of Variance	Cumulative
**1**	14.15284	78.63%	78.63%
**2**	2.14642	11.92%	90.55%
**3**	0.43676	2.43%	92.98%
**4**	0.34949	1.94%	94.92%
**5**	0.24709	1.37%	96.29%
**6**	0.20957	1.16%	97.46%
**7**	0.16178	0.90%	98.36%
**8**	0.09097	0.51%	98.86%
**9**	0.0606	0.34%	99.20%
**10**	0.029	0.16%	99.36%
**11**	0.02881	0.16%	99.52%
**12**	0.02464	0.14%	99.66%
**13**	0.02035	0.11%	99.77%
**14**	0.01659	0.09%	99.86%
**15**	0.01145	0.06%	99.92%
**16**	0.00634	0.04%	99.96%
**17**	0.00457	0.03%	99.98%
**18**	0.00275	0.02%	100.00%

upplementary table S5. Showing eigenvector magnitudes in terms of coefficients of PC1 and PC2

Traits	Coefficients of PC1	Coefficients of PC2
Chlorophyll b	0.25578	-0.09218
Chlorophyll a	0.25242	-0.09097
Caretonoids	0.25023	-0.00819
Proteins	0.2389	-0.14945
Phenols	0.20224	0.41054
Sugars	0.25548	-0.07415
Flavonoids	0.212	0.28587
Lipids	0.2549	-0.02115
Proline	0.21609	0.37881
IAA	0.25129	-0.07506
GA	0.20147	-0.3876
ABA	-0.20738	0.35533
SA	0.22886	0.31314
AA	0.22343	0.32322
Peroxidase	0.2614	-0.00574
Anti-oxidant	0.20799	-0.24583
Catalase	0.25525	-0.02533
H_2O_2	-0.25023	0.12391

Supplementary Table S6. Sowing PCA score data of various treatments for PC1 and PC2

Score data		
**Treatments**	**PC 1 (78.63%)**	**PC 2 (11.92%)**
Control	-4.01419	-2.33367
Control	-3.86228	-2.10401
Control	-3.82553	-1.88563
WA	-0.56671	-1.52915
WA	-0.30993	-1.67931
WA	-0.09062	-1.97748
TG	0.82015	-1.29004
TG	0.90813	-0.99156
TG	0.94571	-1.19099
TL3	1.80414	-0.7338
TL3	2.32343	-1.11938
TL3	2.11261	-0.88526
WA+TG+TL3	6.22263	-0.66914
WA+TG+TL3	6.92333	-0.81971
WA+TG+TL3	6.42563	-0.49739
PEG	-6.56831	0.79545
PEG	-6.66751	0.95387
PEG	-6.98563	0.54012
WA+PEG	-2.30702	0.60417
WA+PEG	-2.01571	0.66049
WA+PEG	-2.18186	0.49631
TG+PEG	-1.35946	1.24074
TG+PEG	-1.07229	1.62258
TG+PEG	-0.84232	1.83586
TL3+PEG	-0.23479	2.1071
TL3+PEG	0.33005	2.46374
TL3+PEG	-0.03186	2.24214
WA+TG+TL3+PEG	4.64728	1.29525
WA+TG+TL3+PEG	4.80574	1.57268
WA+TG+TL3+PEG	4.66718	1.27602
